# Exosomes in epilepsy: bridging neuroinflammation, diagnosis, and therapeutic delivery

**DOI:** 10.3389/fimmu.2025.1667122

**Published:** 2025-09-25

**Authors:** Bin Li, Yanping Zhu, Lixia Chen, Jiajia Cui, Zhenchang Zhang

**Affiliations:** ^1^ Department of Neurology, The Second Hospital & Clinical Medical School, Lanzhou University, Lanzhou, China; ^2^ Department of Respiratory, The Second Hospital & Clinical Medical School, Lanzhou University, Lanzhou, China

**Keywords:** epilepsy, exosomes, blood-brain barrier, neuroinflammation, drug-resistance, drug delivery

## Abstract

Epilepsy, as a chronic neurological disorder marked by recurrent seizures, is closely linked to neuroinflammation and immune dysregulation. Exosomes, extracellular vesicles with potent immunomodulatory properties, have emerged as key players in mitigating epilepsy-associated inflammation by targeting glial activation and balancing pro- and anti-inflammatory cytokine release. Their ability to cross the blood-brain barrier (BBB) enables targeted delivery of anti-inflammatory cargo, such as miRNAs and proteins, offering promise for diagnosing and treating drug-resistant epilepsy. This review highlights exosomes’ dual role as biomarkers of inflammatory pathways and therapeutic vehicles for immune modulation. By suppressing neuroinflammation and restoring neuronal homeostasis, exosome-based strategies may revolutionize epilepsy management, though clinical translation requires further optimization of isolation and engineering techniques.

## 1 Introduction

Epilepsy is a chronic neurological disorder characterized by transient brain dysfunction caused by recurrent, sudden, and excessive hypersynchronous discharges of cortical neurons (1). Accumulating evidence implicates localized neuroinflammation within specific regions of the central nervous system (CNS) as a hallmark pathological feature (2). During seizures, glutamate receptor overactivation, oxidative stress, and elevated pro-inflammatory cytokines disrupt the integrity of the blood–brain barrier (BBB), which facilitates both central and peripheral immune cell infiltration, thereby intensifying seizure susceptibility (3). Among emerging regulatory elements, exosomes have garnered attention for their anti-inflammatory capacity (4, 5). Through suppressing glial cell activation, modulating immune responses, and dampening neuronal excitability, exosomes hold promise in attenuating seizure initiation. Their intrinsic lipid bilayer and capacity to cross the BBB further underscore their translational potential as therapeutic delivery platforms in epilepsy (6).

Despite advances in diagnostics and pharmacotherapy, up to 25% of cases are misdiagnosed (7), and nearly 30% of patients suffer from drug-refractory epilepsy (DRE) (8), reflecting an urgent need for alternative interventions. Although seizure control is achieved in approximately 70% of individuals via monotherapy or polytherapy, the remainder often progresses to DRE, which markedly elevates disease burden and mortality (9). Notably, compromised BBB function has been closely linked to antiepileptic drug resistance (10), and enhancing drug penetration across the BBB may significantly improve therapeutic efficacy (11). With growing recognition of their therapeutic and diagnostic utility, cs have become a focal point in epilepsy research (12). This review outlines the roles of exosomes in modulating epilepsy-associated neuroinflammation, highlights their mechanistic involvement in seizure suppression, and evaluates their utility in overcoming drug resistance, offering a conceptual framework for next-generation epilepsy management.

## 2 Overview of exosomes

Exosomes are nanoscale extracellular vesicles (EVs), typically 50 – 150 nm in diameter, that encapsulate a rich cargo of nucleic acids, proteins, and lipids (13, 14). They are secreted by virtually all cell types and are detectable in a variety of biological fluids, including blood, urine, and cerebrospinal fluid (15, 16). Far from being inert cellular byproducts, exosomes have emerged as dynamic mediators of intercellular communication, orchestrating diverse physiological and pathological responses to environmental cues (17). In the CNS, exosomes have gained increasing attention for their multifaceted roles in neurodevelopment, synaptic plasticity, and immune surveillance (18). Their pathological relevance is underscored by mounting evidence implicating them in the onset and progression of various neurological diseases (19). For example, microglia-derived exosomal miR-124-3p has been shown to exert neuroprotective effects by targeting the Rela/ApoE signaling axis, thereby mitigating neuronal damage and neuroinflammation (20). This axis is critical, as RELA (p65), a subunit of NF-κB, modulates inflammatory gene expression, while ApoE is involved in lipid transport and neural repair. In major depressive disorder (MDD), Kuwano et al. (21) identified exosomes enriched with IL - 34 and the tetraspanin CD81 as potential diagnostic biomarkers. IL - 34 is known to promote microglial survival and neurogenesis, suggesting that exosomal IL - 34/CD81 signatures may reflect active neuroinflammatory or neurotrophic processes in MDD. Exosomes also function as vectors for therapeutic repair. In a rat model of focal cerebral ischemia, systemically administered exosomes were shown to localize to the ischemic brain, where they fuse with recipient cells, deliver functional biomolecules, and promote neuroregeneration (22). Mechanistically, this may involve the modulation of PI3K/AKT and MAPK signaling cascades, which are central to cell survival and axonal remodeling (23). Moreover, exosomes exhibit anti-inflammatory potential in perinatal brain injury by suppressing pro-inflammatory cytokine production and fostering a reparative microenvironment through the delivery of anti-inflammatory miRNAs and immunomodulatory proteins such as TGF-β (24). Emerging studies also suggest that exosomes derived from brain-metastatic cancer cells can precondition the brain microenvironment to support tumor growth (25). Together, these studies underscore the dualistic nature of exosomes in the CNS, as both mediators of repair and drivers of pathology, highlighting their potential as diagnostic biomarkers and therapeutic targets across a broad spectrum of neurological conditions.

## 3 Exosomes in epilepsy-associated neuroinflammation

### 3.1 Astrocytes and exosomes

Glial cell activation is a hallmark of neuroinflammatory responses in epilepsy (26). Astrocyte proliferation is often more persistent than microglial activation, contributing to more sustained inflammatory damage (27, 28). Astrocytes perform diverse roles, including neuroprotection, neurotransmitter regulation, extracellular ion buffering, synaptic signaling, and maintenance of the BBB (29). Upon CNS injury, reactive astrocytosis occurs, accompanied by glutamate excitotoxicity, altered gap junctions, and mitochondrial dysfunction. Mesenchymal stem cell (MSC)-derived exosomes alleviate pilocarpine-induced epilepsy in mice by reducing expression of glial fibrillary acidic protein (GFAP) and complement component 3 (C3) in bilateral hippocampi, dampening IL - 1β and TNF-α secretion, and mitigating intracellular Ca²^+^ influx (30). These changes were associated with improved spatial learning and memory, indicating a restoration of astrocytic function and mitochondrial homeostasis. The underlying mechanism involves the modulation of the Nrf2/NF-κB signaling axis, where Nrf2 suppresses oxidative stress-induced inflammation, while NF-κB promotes glial proliferation and cytokine release (31). Exosomes appear to upregulate Nrf2 nuclear translocation while inhibiting NF-κB activity, thus attenuating reactive astrocyte activation (30).

In particular, MSC-derived exosomes suppress the formation of neurotoxic A1 astrocytes, induced by pro-inflammatory cytokines such as IL - 1α, TNF-α, and C1q, by downregulating C3, a key molecular marker of the A1 phenotype (32). Inhibition of the C3-C3aR signaling axis by exosomal miRNAs and proteins attenuates synaptotoxic effects and neuronal death (33). Moreover, exosomes modulate STAT3 phosphorylation, a pathway implicated in A2 astrocyte differentiation and neuroprotection, thus promoting a phenotypic shift toward anti-inflammatory, pro-repair states (34–36). Targeting reactive astrocytes, particularly the neurotoxic A1 phenotype, offers a promising therapeutic strategy in epilepsy (37). A1 astrocytes, which are induced during inflammation, disrupt synapses and exhibit neurotoxicity (38, 39). Exosomes not only suppress proliferation of A1 astrocytes but may also promote their phenotypic reversion to non-reactive states via Nrf2-NF-κB signaling (40). In addition to molecular inhibition of A1 activation, exosomes restore cellular metabolism by improving mitochondrial membrane potential and reducing ROS, thereby promoting phenotypic reversion to homeostatic astrocytes (41). Furthermore, MSC-derived exosomes restore mitochondrial membrane potential, reduce Ca²^+^ influx, and reverse abnormal calcium signaling in hippocampal astrocytes (42–44). Compared to MSCs, their exosomes—containing anti-inflammatory mRNA, miRNA, and proteins, exhibit lower immunogenicity, greater stability, and ease of storage, making them ideal vectors for targeted drug delivery to astrocytes within the hippocampus (45).

### 3.2 Microglia and exosomes

The degree of microglial activation is positively correlated with the duration and severity of epilepsy (46). As key immune cells in the brain, microglia orchestrate inflammatory responses, which are implicated in neurodegeneration, hippocampal inflammation, and BBB disruption following status epilepticus (SE) (47). Long et al. (45) showed that A1-type MSC-derived exosomes administered after SE reduce neurodegeneration, dampen hippocampal inflammation, and preserve neurogenesis and cognitive function. Elevated glutamate levels and persistent neuroinflammation following SE are major contributors to excitotoxicity and neuronal apoptosis (48, 49). Moreover, GABAergic inhibition is suppressed during seizures, disrupting the excitatory-inhibitory balance. Exosomes help restore this balance by directly protecting GABAergic neurons through multiple mechanisms, including suppression of microglial activation and modulation of cytokine profiles (50, 51). Specifically, exosomes downregulate pro-inflammatory cytokines such as TNF-α and MCP - 1 while upregulating IL - 10, an anti-inflammatory cytokine that promotes neuronal survival (52, 53). This shift in the cytokine milieu reduces oxidative stress, attenuates neuroinflammation, and limits excitotoxic injury to GABAergic interneurons, which are essential for seizure containment (54, 55). While resting microglia aid in debris clearance and neuroprotection, their hyperactivation leads to the release of pro-inflammatory and cytotoxic agents that drive neuronal loss (56). In SE mice, A1 exosomes attenuated microglial activation and suppressed pro-inflammatory cytokines (TNF-α, MCP - 1) while enhancing anti-inflammatory mediators (IL - 10, IL - 6). These changes were associated with the preservation of GABAergic neurons, reduced apoptotic markers, and improved synaptic integrity (45). Inflammatory oxidative stress in the hippocampus exacerbates cognitive deficits and memory loss via ROS-induced synaptic dysregulation (57, 58). Thus, A1 exosomes, by maintaining GABA-glutamate homeostasis, suppressing microglial overactivation, and preserving interneurons, attenuate SE-induced neurodegeneration and prevent progression to chronic epilepsy (45).

### 3.3 Oligodendrocytes and exosomes

Oligodendrocytes serve as the principal myelinating cells within the central nervous system (CNS), playing an indispensable role in maintaining neuronal conductivity and structural integrity (59). In pathological contexts characterized by demyelination, oligodendrocyte viability is compromised, particularly due to inflammation-induced apoptosis, which severely impairs the capacity for remyelination and functional recovery (60). Notably, demyelinating lesions are frequently observed in individuals with epilepsy, a phenomenon that may arise from disrupted autonomic regulation and altered neuronal-glial dynamics (61). Low-dose interferon-γ (IFN-γ) stimulation of dendritic cells induces the secretion of exosomes enriched with remyelination-associated microRNAs, including miR-219, miR-335, and miR-494-3p (62–64). These exosomes exhibited preferential tropism for oligodendrocytes, followed sequentially by uptake in microglia and astrocytes, suggesting a degree of cell-specific targeting that holds translational relevance for therapeutic interventions in demyelinating diseases (65, 66). Mechanistically, dendritic cell–derived exosomes have been shown to modulate the expression of pivotal oligodendrocyte lineage regulators, including NeuroD1, PDGFRα, and ELOVL7, predominantly via miR-219-mediated gene regulation (67). Continued exploration of these exosome–oligodendrocyte interactions may yield novel strategies for enhancing remyelination in epilepsy and related demyelinating diseases.

### 3.4 Exosomes and the immune response

The immune system exerts a pivotal influence on the onset and progression of epilepsy (68). Inflammatory responses represent transient manifestations of immune activation, encompassing both pro- and anti-inflammatory mediators. Infiltrating immune cells release cytokines such as TNF-α and IL - 18, thereby amplifying neuroinflammation (69); conversely, anti-inflammatory cytokines like IL - 10 contribute to the resolution of the immune response (70). Under physiological conditions, the blood–brain barrier (BBB) maintains neural immune privilege by restricting peripheral immune cell infiltration. However, during inflammation, endothelial dysfunction compromises BBB integrity and enhances its permeability, subsequently lowering neuronal firing thresholds and heightening seizure susceptibility. While moderate inflammation may facilitate central nervous system repair and synaptic remodeling, sustained or dysregulated immune activation promotes epileptogenesis. The convergence of BBB disruption and increased neuronal excitability constitutes a fundamental axis in the pathophysiology of epilepsy. Exosomes actively participate in immune regulation through mechanisms such as antigen presentation (71), immune suppression (72), immune surveillance (73), and intercellular signaling. For instance, MSC-derived exosomes upregulate IL - 10 and other immunosuppressive molecules, thereby promoting Tregs proliferation and conferring potent immunomodulatory effects.

## 4 Exosomes and their cargo as potential biomarkers in epilepsy

Exosomes derived from various cell types encapsulate proteins, mRNAs, and miRNAs (74), reflecting the diverse and complex nature of their molecular cargo. This heterogeneity forms the foundation for their utility as biomarkers in epilepsy (75). Notably, the dysregulation of specific miRNAs is closely associated with epileptogenesis. For instance, miR-23b-3p, a critical regulator of neuronal excitability, is markedly reduced in epilepsy, and its loss can precipitate fatal seizures (76). Alterations in circulating exosomal miRNAs have been observed in patients with epilepsy. Emerging evidence from serum miRNA profiling has revealed marked dysregulation of circulating microRNAs in individuals with epilepsy compared to healthy controls. Notably, several miRNAs, including miR-27a-3p, miR-181a-5p, miR-134, miR-221, miR-155, and miR-146—are significantly upregulated, whereas others such as miR-132, miR-125a-5p, and miR-34c-5p exhibit reduced expression levels (77). Besides, miR-106b-5p demonstrated a sensitivity of 80% and specificity of 81%, suggesting strong potential as a novel diagnostic biomarker. Beyond circulating miRNAs, exosomal dynamics have also garnered attention as pathophysiological hallmarks of epileptogenesis. Batool et al. (78) reported that elevated levels of exosomal secretion persisted in epileptic mice two weeks after status epilepticus (SE), suggesting a sustained involvement of exosomes in the chronic phase of epilepsy.

## 5 Exosomes in epilepsy therapy

### 5.1 Exosomes suppress neuroinflammation

**Figure 1:**
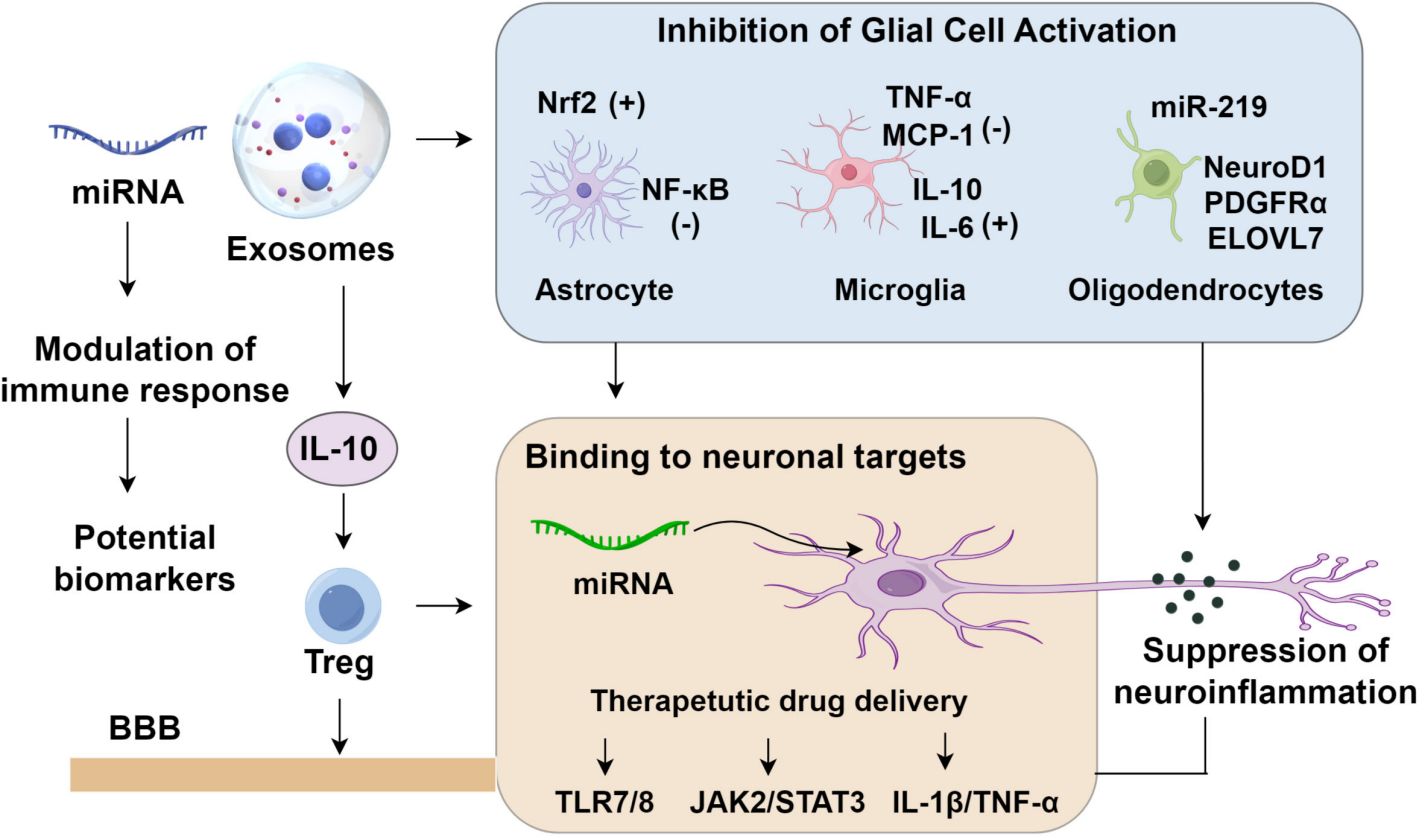
Roles of exosomes in epilepsy.

Inflammation serves both as an initiator and a consequence of epileptic seizures, forming a self-perpetuating loop that amplifies seizure frequency and severity. Seizure activity induces excessive glutamate receptor activation, oxidative stress, and elevated levels of pro-inflammatory cytokines, all of which compromise the integrity of the BBB. Mounting evidence implicates exosomes as key modulators of neuroinflammatory processes. These vesicles mitigate glial activation, modulate immune signaling, suppress neuronal hyperexcitability, selectively interact with neuronal targets, and limit neuronal loss (79). Notably, exosomes derived from epileptogenic tubers in tuberous sclerosis complex were investigated using small RNA sequencing, revealing microRNAs enriched within these vesicles that are capable of activating toll-like receptors TLR7/8 (80). This activation initiated a neuroinflammatory cascade, substantially upregulating pro-inflammatory cytokines and heightening seizure susceptibility, thereby identifying a novel therapeutic target for drug-resistant epilepsy associated with tuberous sclerosis (80). Intravenous administration of astrocyte-derived exosomes has been shown to attenuate microglial activation in epileptic rats by suppressing the JAK2/STAT3 signaling pathway, thereby mitigating neuroinflammation and neuronal apoptosis and contributing to effective seizure control (81–83). In a separate study, mesenchymal stem cell (MSC)-derived exosomes delivered via intracerebroventricular injection in pilocarpine-induced status epilepticus (SE) mice promoted astrocyte proliferation and alleviated neuroinflammatory responses, resulting in improved cognitive performance. Consistently, bone marrow MSC-derived exosomes were reported to rapidly accumulate in the hippocampus following muscarine-induced SE, highlighting their capacity for targeted delivery to affected brain regions (45). The treatment preserved glutamatergic and GABAergic neurons, alleviated hippocampal inflammation, and ameliorated SE-induced cognitive impairments. Collectively, these findings illustrate the multifaceted therapeutic potential of exosomes in epilepsy through neuroinflammatory modulation and cognitive restoration, offering promising avenues for drug-resistant epilepsy (**Figure 1**).

### 5.2 Neuroprotective functions of exosomes

MSC-derived exosomes serve as pivotal mediators of neural repair in epilepsy, exerting their effects through four principal mechanisms: first, directly enhancing the survival and differentiation of damaged neurons; second, indirectly attenuating glial activation, thereby mitigating neuroinflammation and oxidative stress while promoting neurovascular regeneration; third, restoring systemic metabolic homeostasis; and fourth, modulating immune responses via the secretion of cytokines and the presentation of distinctive membrane-associated molecules (84). Exosomes derived from bone marrow MSCs have been shown to confer robust neuroprotection in epilepsy models. One study (85) reported that these exosomes significantly alleviated neuronal loss in the dentate gyrus and CA1 regions of the hippocampus in mice with status epilepticus. Supporting these findings, intranasal administration of MSC-derived exosomes enabled effective hippocampal targeting, reduced brain injury, preserved neurogenesis, and sustained cognitive performance (86). Importantly, this approach also suppressed pro-inflammatory cytokine-induced iNOS expression, thereby mitigating neuronal damage. Complementarily, systemic injection of microglia-derived exosomes in a transient middle cerebral artery occlusion model reduced brain atrophy, improved neuronal function, promoted oligodendrocyte regeneration, and ameliorated white matter injury (87). These therapeutic effects were mediated through intercellular signaling pathways that modulate inflammation and coordinate immune responses to facilitate neural repair. Collectively, this body of evidence underscores the multifaceted neuroprotective roles of MSC-derived exosomes and illuminates their translational potential for epilepsy therapy.

### 5.3 Exosomes as drug delivery vehicles in epilepsy therapy

Current epilepsy management primarily relies on antiepileptic drugs (AEDs) such as phenytoin, carbamazepine, and valproate. Despite the availability of approximately 30 AEDs targeting diverse molecular mechanisms, clinical challenges persist, including drug resistance (88) and adverse effects (89). Nearly one-third of patients remain refractory to current therapies. One critical barrier in AED efficacy is the BBB. Exosomes, owing to their dual capacity to participate in neuroimmune communication and traverse the BBB via receptor-mediated endocytosis or fusion, hold significant promise for targeted CNS drug delivery. Moreover, exosomes can bypass the P-glycoprotein efflux system, enabling stable and sustained drug delivery across the BBB (90). Exosomes serve as versatile vectors for delivering proteins, small and large molecules, and nucleic acids (91). Their intrinsic targeting capability, driven by membrane proteins and glycan structures that recognize specific receptors on recipient cells, enables precise delivery. Additionally, exosomes can protect therapeutic agents from enzymatic degradation and ensure efficient BBB penetration and tissue-specific accumulation. Various engineering techniques such as surface modification, transfection, electroporation, ultrasound, extrusion, and freeze–thaw cycles have been developed to enhance drug encapsulation and delivery.

Previous studies have predominantly focused on the role of endogenous exosomal miRNAs in modulating neuroinflammation in epilepsy (30, 45). Although clinical studies using exosomes as drug carriers for epilepsy remain lacking, advances from other neurodegenerative disorders offer instructive parallels. For example, Haney et al. (92) successfully delivered catalase-loaded exosomes intranasally, achieving significant CNS accumulation and neuroprotection in murine models without provoking immune rejection. Curcumin, known for its antioxidant, anti-inflammatory, lipid-lowering, and anti-aggregatory effects (93), suffers from poor bioavailability due to low absorption and rapid metabolism. Kalani et al. (94) encapsulated curcumin into embryonic stem cell–derived exosomes, enhancing its bioavailability and reducing glial fibrillary acidic protein expression, thereby limiting astrogliosis. Dad et al. (95) proposed an innovative strategy for treating post-epileptic depression by transfecting miR-219 and miR-338 into synthetic polyvalent antibodies and utilizing exosomes as carriers. This approach effectively suppressed immune responses while promoting axonal regeneration and remyelination in the injured CNS. Extending this concept, researchers proposed loading therapeutic mRNA, miRNA, and proteins into exosomes to enhance their capacity to traverse the blood–brain barrier, thereby offering adjunctive benefits in the aftermath of status epilepticus by reducing seizure burden (96). Moreover, brain-derived exosomes carrying acid sphingomyelinase (ASM) or functional miRNAs have been shown to modulate neuronal excitability through targeted regulation of gene expression and signaling pathways (97), providing a conceptual framework for precision-targeted epilepsy interventions. In summary, exosomes represent a powerful drug delivery platform for addressing drug-resistant epilepsy. Their ability to traverse the BBB and deliver therapeutic agents directly to target tissues underscores their potential to transform epilepsy treatment paradigms.

## 6 Conclusion

In summary, exosomes represent a transformative avenue for epilepsy diagnosis and therapy, leveraging their innate ability to modulate neuroinflammation, cross the BBB, and deliver bioactive cargo. By suppressing glial activation, regulating immune responses, and serving as biomarkers or drug carriers, exosomes address critical gaps in managing drug-resistant epilepsy. Specifically, exosomal miRNAs such as miR-124, miR-219, and miR-146a have demonstrated significant promise in experimental models due to their ability to suppress neuroinflammation, promote remyelination, and modulate microglial and astrocytic activity. These molecules merit further investigation as potential therapeutic candidates.

Nonetheless, several key engineering and translational hurdles remain. These include the development of scalable and reproducible exosome isolation techniques, standardization of cargo loading methods, real-time tracking of *in vivo* distribution, and improving cell-type-specific targeting to minimize off-target effects. Future research must prioritize mechanistic validation of exosomal miRNAs/proteins, optimization of delivery systems, and rigorous clinical trials to harness their full potential. Addressing these challenges will be essential to position exosome-based strategies as a cornerstone of precision medicine in epilepsy and related neuroinflammatory disorders.
